# For “Durin’ the War”

**Published:** 1896-06

**Authors:** 


FOR "DURIN' THE WAR."—The editor of the Red Back led the *forlorn hope* at Ft. Worth in the battle for professional honor and self-respect, in opposing the recognition of the homeopathic and eclectic species of the genus quack. The bill will go before the legislature simply asking for a board of physicians, "recognizing homeopaths only so far as the Constitution recognizes them."* If the legislature shall force us to accept a mixed board that is a very different thing from our asking for it. But why should the legislature not give us a board of regular physicians, and then create a board of homeopaths, if they wish a board? The writer's idea is, to ignore homeopaths entirely; to not consider them as physicians any more than the old lady with her teas; unless they attempt to practice by what they call the "allopathic system"; then require examination or prosecute them for practicing in violation of law. Do not require them to be examined, nor licensed. What have we to do with their pathies? It is not medicine. So long as they practice homeo pathy let them alone. The JOURNAL is enlisted for the war; not only to oppose this species, but all other quacks,—in the profession as well as out;—for there are quacks who have diplomas. But there are diplomas of many kinds. A diploma from the "Texas Health College" alleged to be at Mound City, Texas, nor one from the Chicago concern, mentioned in this issue, will not afford any protection to the holder, nor entitle him to practice medicine; for it is the decision of the attorney general that notwithstanding these concerns claim to

---

*See "Resolution," elsewhere.

be "regularly chartered institutions" they are not such as contemplated by the law; a college that does not exist, and never did exist, can not issue a diploma—although a charter may have been granted for such college; and the JOURNAL wants to say—here and now—that wherever any of our readers hear of a person practicing medicine by virtue of a diploma from either of these—or any other bogus medical college—if they will send us the facts, we will personally see the attorney general and have him instruct the county or district attorney to arrest and prosecute such persons.

But that really is unnecessary. The attorney general says that although these colleges were chartered, they never existed, and the so-called diploma is a fraud, and any one practicing by virtue of having such diploma, notwithstanding it is on record, is notoriously practicing in violation of the law. Any citizen, therefore, may file information with the county attorney, if the grand jury is not in session; (it is not necessary to wait for the grand jury) and the attorney is bound by law to have a warrant issued for the arrest of the party, and in default of bond for appearance at court, send him to the lockup.

While a physician will naturally shrink from the performance of such a duty,—"for fear of having his motives questioned,"—let him ask himself the question; is it not obligatory, a duty you should not shirk, knowing that the law is being violated to the danger and detriment of the public health, to report it? I should not hesitate to do so, and I wouldn't care a red cent what Tom or Dick thought was my motive. And the JOURNAL urges upon its readers to take this step. The profession is clamoring for a law to protect the public from illegitimate practitioners, yet here is a case at your door, and here is a law to meet it; how can you, from motives of false delicacy, refuse to ask that the law, such as it is, shall be enforced? At any rate we want to locate every "Texas Health College diploma" in Texas, and get the name of the holder, and if the local profession will not move, we will, as stated, get instructions from the attorney general to the local attorney to prosecute.

And, by the bye, this reminds us that the notorious Orin Robinson, the alleged founder of that famous myth of Mound City, the "Texas Health College" (why not the Texas sickness college?) is perambulating around in North Texas, about Fannin and Lamar county.

